# Hand Cleanliness

**Published:** 1900-04

**Authors:** 


### HAND CLEANLINESS.

When we read some of the descriptions of the methods proposed by surgeons and bacteriologists for the proper cleansing of the hands, we are sometimes rather apt to feel that life is somewhat short and uncertain, and that a pressing case might pass out of our hands into the undertaker's before we might be through with such complicated ablutions. If that is the case, there is only one thing left to do—use rubber gloves that may be boiled in a few minutes. But we are not inclined to think that the proper washing of the hands is such a laborious procedure after all, and those of us who prune our fellow mortals with any degree of frequency, know that the habit of washing our hands thoroughly and conscientiously is one that is easy to acquire. Whichever plan we adopt, it is doubtful, whether more than fifteen or twenty minutes need be employed in the process, and there can be no question that it is time very well spent. No man in the world can afford so little as the surgeon to take useless chances, and the comfort taken by a few specimens of humanity in dirty finger nails is too dearly bought at the cost of human lives. The true surgeon is careful and methodical, for he knows that every operation consists in three stages; preparation, performance, and after-care. The surgeon who has not drilled himself into taking equal pains with each one of these three steps will remain a bungler until he has changed the tenor of his ways, and will remain, however brilliant he may be as an operator, inferior to the careful, methodical man who leaves nothing to luck.—*International Jour. of Surgery.*

